# Supplementary figures and images for: Risk factors for COVID-19 mortality among telehealth patients in Bangladesh: A prospective cohort study
Source: PLOS Glob Public Health. 2023 Jun 14;3(6):e0001971. doi: 10.1371/journal.pgph.0001971 (PMC10266619; doi:10.1371/journal.pgph.0001971)

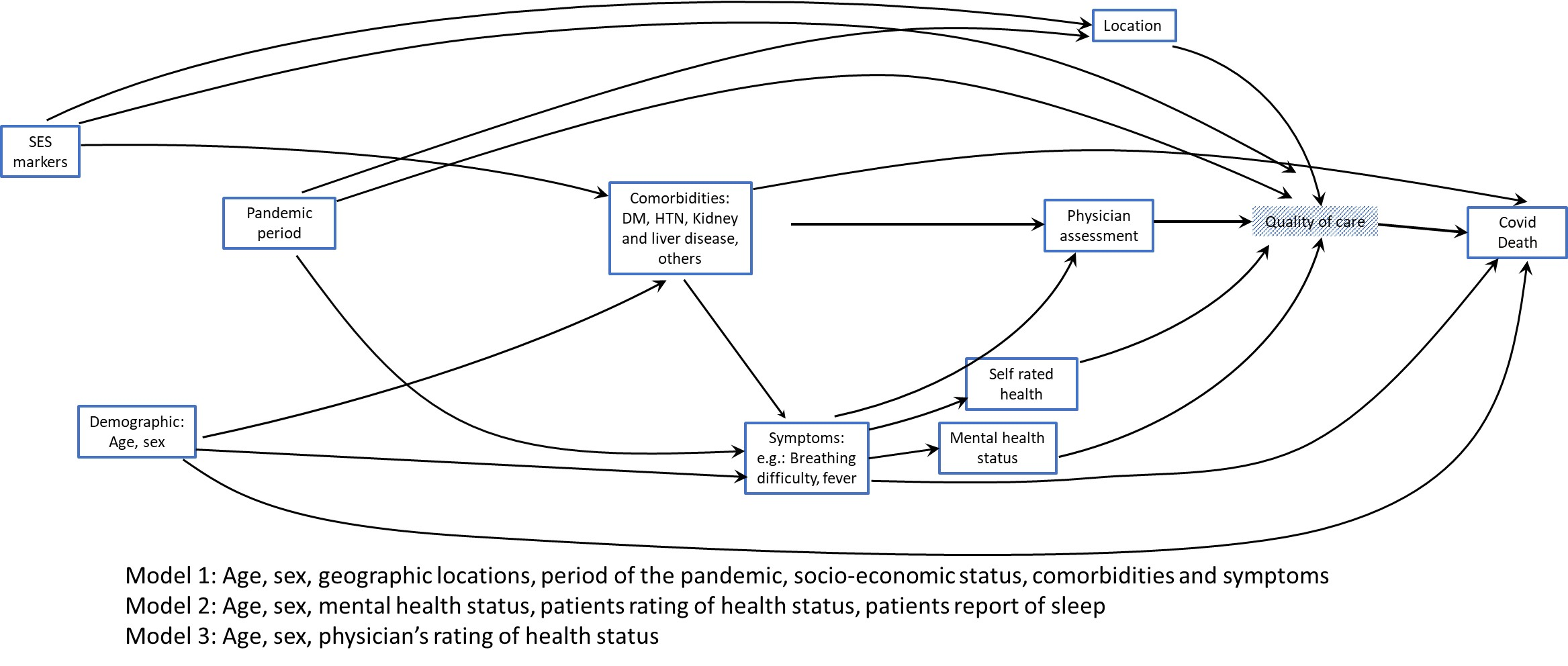

Supplement: S1 Fig — (TIF) [file pgph.0001971.s001.tif]

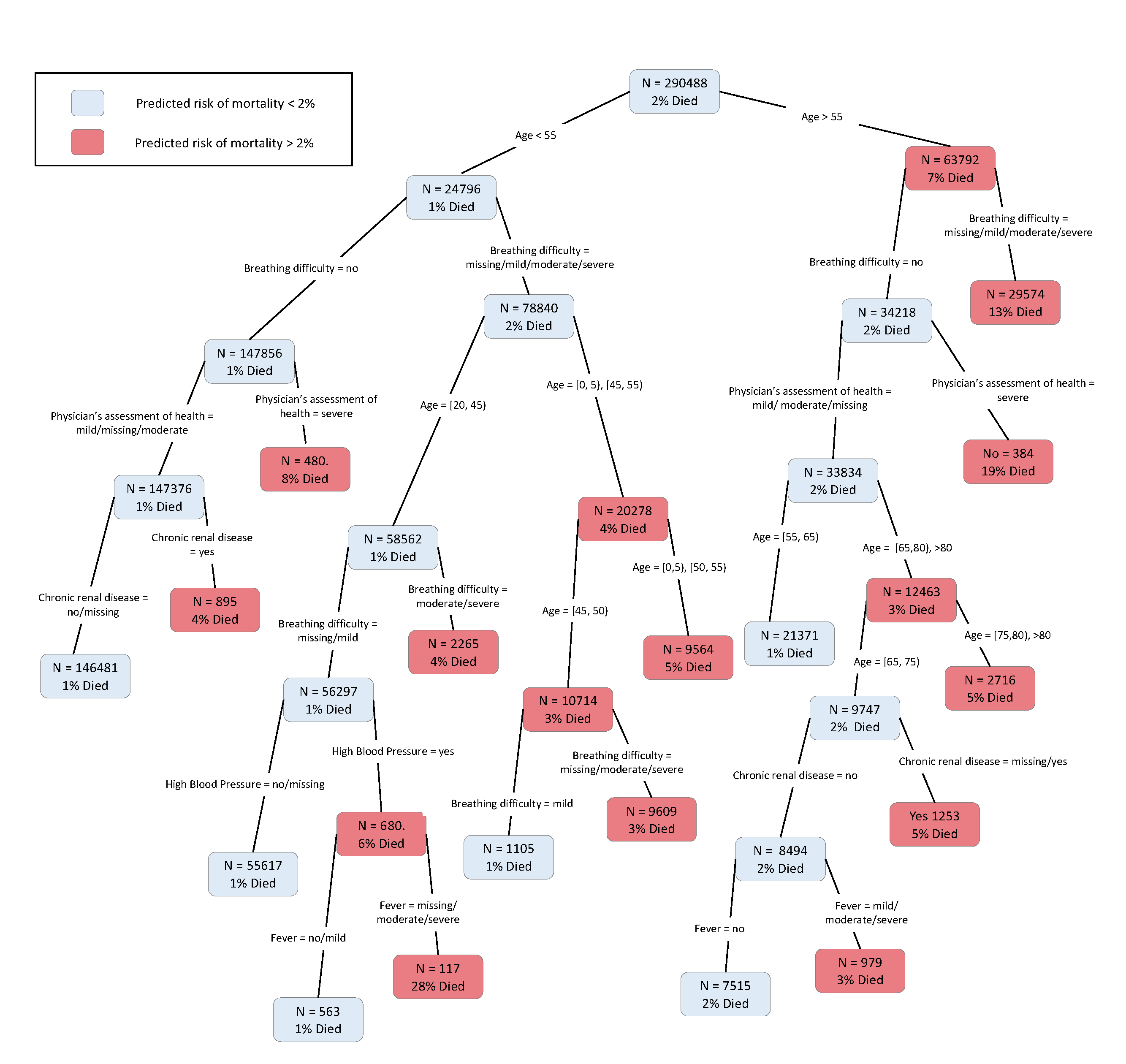

Supplement: S2 Fig — N represents the number of patients in the original data (as opposed to the resampled data used to create the tree). Blue indicates the predicted risk of mortality less than or equal to 2%; red indicates the predicted risk of mortality greater than 2% in the data. (TIFF) [file pgph.0001971.s002.tiff]
