# Supplementary material for: Risk factors for COVID-19 mortality among telehealth patients in Bangladesh: A prospective cohort study
Source: PLOS Glob Public Health. 2023 Jun 14;3(6):e0001971. doi: 10.1371/journal.pgph.0001971 (PMC10266619; doi:10.1371/journal.pgph.0001971)
Supplement: S1 Table — (DOCX) [file pgph.0001971.s003.docx]

|  | Overall (N=326,700) | | Not missing (N=290,488) | | Missing (N=36,212) | |
| --- | --- | --- | --- | --- | --- | --- |
|  | n | % or Mean (SD) | n | % or Mean (SD) | n | % or Mean (SD) |
| Age |  |  |  |  |  |  |
| 20 - 25 | 19,804 | 6.1% | 17,697 | 6.1% | 2,107 | 5.8% |
| 0 - 5 | 2,430 | 0.7% | 2,029 | 0.7% | 401 | 1.1% |
| 5 – 10 | 2,083 | 0.6% | 1,944 | 0.7% | 139 | 0.4% |
| 10 - 15 | 3,621 | 1.1% | 3,292 | 1.1% | 329 | 0.9% |
| 15 – 20 | 12,408 | 3.8% | 9,701 | 3.3% | 2,707 | 7.5% |
| 25 – 30 | 42,699 | 13.1% | 36,061 | 12.4% | 6,638 | 18.3% |
| 30 - 35 | 38,902 | 11.9% | 36,195 | 12.5% | 2,707 | 7.5% |
| 35 – 40 | 44,748 | 13.7% | 38,854 | 13.4% | 5,894 | 16.3% |
| 40 – 45 | 30,389 | 9.3% | 28,543 | 9.8% | 1,846 | 5.1% |
| 45 – 50 | 31,867 | 9.8% | 27,956 | 9.6% | 3,911 | 10.8% |
| 50 – 55 | 25,985 | 8.0% | 24,424 | 8.4% | 1,561 | 4.3% |
| 55 - 60 | 25,780 | 7.9% | 22,720 | 7.8% | 3,060 | 8.5% |
| 60 - 65 | 15,762 | 4.8% | 14,645 | 5.0% | 1,117 | 3.1% |
| 65 - 70 | 15,112 | 4.6% | 13,004 | 4.5% | 2,108 | 5.8% |
| 70 - 75 | 7,325 | 2.2% | 6,733 | 2.3% | 592 | 1.6% |
| 75 - 80 | 4,350 | 1.3% | 3,676 | 1.3% | 674 | 1.9% |
| 80+ | 3,435 | 1.1% | 3,014 | 1.0% | 421 | 1.2% |
| Sex |  |  |  |  |  |  |
| Female | 103,975 | 31.826% | 91,645 | 31.549% | 12,330 | 34.049% |
| Male | 222,725 | 68.174% | 198,843 | 68.451% | 23,882 | 65.951% |
| Region |  |  |  |  |  |  |
| Dhaka | 166,885 | 51.1% | 150,610 | 51.8% | 16,275 | 44.9% |
| Missing | 40,785 | 12.5% | 37,528 | 12.9% | 3,257 | 9.0% |
| Chittagong | 46,154 | 14.1% | 39,412 | 13.6% | 6,742 | 18.6% |
| Mymensingh | 6,890 | 2.1% | 5,814 | 2.0% | 1,076 | 3.0% |
| Barisal | 10,674 | 3.3% | 9,244 | 3.2% | 1,430 | 3.9% |
| Sylhet | 8,466 | 2.6% | 7,387 | 2.5% | 1,079 | 3.0% |
| Khulna | 18,635 | 5.7% | 15,474 | 5.3% | 3,161 | 8.7% |
| Rajshahi | 17,726 | 5.4% | 15,357 | 5.3% | 2,369 | 6.5% |
| Rangpur | 10,485 | 3.2% | 9,662 | 3.3% | 823 | 2.3% |
| Presence of a Caregiver |  |  |  |  |  |  |
| No | 35,412 | 10.8% | 35,398 | 12.2% | 14 | 0.0% |
| Missing | 144,686 | 44.3% | 108,541 | 37.4% | 36,145 | 99.8% |
| Yes | 146,602 | 44.9% | 146,549 | 50.4% | 53 | 0.1% |
| **Change in Behavior after Covid Diagnosis** |  |  |  |  |  |  |
| No | 170,281 | 52.1% | 170,219 | 58.6% | 62 | 0.2% |
| Missing | 139,443 | 42.7% | 103,298 | 35.6% | 36,145 | 99.8% |
| Yes | 16,976 | 5.2% | 16,971 | 5.8% | 5 | 0.0% |
| **Rules at home after Covid Diagnosis** |  |  |  |  |  |  |
| Isolation |  |  |  |  |  |  |
| No | 90,887 | 27.8% | 90,853 | 31.3% | 34 | 0.1% |
| Missing | 143,324 | 43.9% | 107,181 | 36.9% | 36,143 | 99.8% |
| Yes | 92,489 | 28.3% | 92,454 | 31.8% | 35 | 0.1% |
| Doctor Physical Examination |  |  |  |  |  |  |
| Mild | 159,538 | 48.8% | 159,486 | 54.9% | 52 | 0.1% |
| Missing | 134,854 | 41.3% | 98,713 | 34.0% | 36,141 | 99.8% |
| Severe | 2,471 | 0.8% | 2,468 | 0.8% | 3 | 0.0% |
| Moderate | 29,837 | 9.1% | 29,821 | 10.3% | 16 | 0.0% |
| Distance with Family |  |  |  |  |  |  |
| No | 26,755 | 8.2% | 26,737 | 9.2% | 18 | 0.0% |
| Missing | 160,860 | 49.2% | 124,712 | 42.9% | 36,148 | 99.8% |
| Yes | 139,085 | 42.6% | 139,039 | 47.9% | 46 | 0.1% |
| Masking |  |  |  |  |  |  |
| No | 47,479 | 14.5% | 47,457 | 16.3% | 22 | 0.1% |
| Missing | 160,860 | 49.2% | 124,712 | 42.9% | 36,148 | 99.8% |
| Yes | 118,361 | 36.2% | 118,319 | 40.7% | 42 | 0.1% |
| Isolation |  |  |  |  |  |  |
| Yes | 155,156 | 47.5% | 155,097 | 53.4% | 59 | 0.2% |
| No | 23,260 | 7.1% | 23,247 | 8.0% | 13 | 0.0% |
| Missing | 148,284 | 45.4% | 112,144 | 38.6% | 36,140 | 99.8% |
| **Self-rated Patient Health** |  |  |  |  |  |  |
| Enough Sleep |  |  |  |  |  |  |
| No | 21,961 | 6.7% | 21,941 | 7.6% | 20 | 0.1% |
| Missing | 136,975 | 41.9% | 100,835 | 34.7% | 36,140 | 99.8% |
| Yes | 167,764 | 51.4% | 167,712 | 57.7% | 52 | 0.1% |
| Health Improvement |  |  |  |  |  |  |
| No | 12,721 | 3.9% | 12,710 | 4.4% | 11 | 0.0% |
| Missing | 137,648 | 42.1% | 101,506 | 34.9% | 36,142 | 99.8% |
| Yes | 176,331 | 54.0% | 176,272 | 60.7% | 59 | 0.2% |
| **Comorbidities** |  |  |  |  |  |  |
| Chronic respiratory illness |  |  |  |  |  |  |
| No | 179,640 | 55.0% | 179,571 | 61.8% | 69 | 0.2% |
| Missing | 137,099 | 42.0% | 100,958 | 34.8% | 36,141 | 99.8% |
| Yes | 9,961 | 3.0% | 9,959 | 3.4% | 2 | 0.0% |
| Diabetes |  |  |  |  |  |  |
| No | 152,814 | 46.8% | 152,757 | 52.6% | 57 | 0.2% |
| Missing | 137,099 | 42.0% | 100,958 | 34.8% | 36,141 | 99.8% |
| Yes | 36,787 | 11.3% | 36,773 | 12.7% | 14 | 0.0% |
| High Blood Pressure |  |  |  |  |  |  |
| No | 153,277 | 46.9% | 153,222 | 52.7% | 55 | 0.2% |
| Missing | 137,099 | 42.0% | 100,958 | 34.8% | 36,141 | 99.8% |
| Yes | 36,324 | 11.1% | 36,308 | 12.5% | 16 | 0.0% |
| Kidney Disease |  |  |  |  |  |  |
| No | 186,383 | 57.1% | 186,313 | 64.1% | 70 | 0.2% |
| Missing | 137,099 | 42.0% | 100,958 | 34.8% | 36,141 | 99.8% |
| Yes | 3,218 | 1.0% | 3,217 | 1.1% | 1 | 0.0% |
| Chronic Liver Disease |  |  |  |  |  |  |
| No | 188,365 | 57.7% | 188,294 | 64.8% | 71 | 0.2% |
| Missing | 137,099 | 42.0% | 100,958 | 34.8% | 36,141 | 99.8% |
| Yes | 1,236 | 0.4% | 1,236 | 0.4% | 0 | 0.0% |
| Other |  |  |  |  |  |  |
| No | 176,868 | 54.1% | 176,806 | 60.9% | 62 | 0.2% |
| Missing | 137,099 | 42.0% | 100,958 | 34.8% | 36,141 | 99.8% |
| Yes | 12,733 | 3.9% | 12,724 | 4.4% | 9 | 0.0% |
| **Physician Assessment** |  |  |  |  |  |  |
| Mental Health Status |  |  |  |  |  |  |
| No | 161,365 | 49.4% | 161,312 | 55.5% | 53 | 0.1% |
| Missing | 141,245 | 43.2% | 105,099 | 36.2% | 36,146 | 99.8% |
| Yes | 24,090 | 7.4% | 24,077 | 8.3% | 13 | 0.0% |
| Physically Challenged |  |  |  |  |  |  |
| No | 181,342 | 55.5% | 181,279 | 62.4% | 63 | 0.2% |
| Missing | 142,543 | 43.6% | 106,396 | 36.6% | 36,147 | 99.8% |
| Yes | 2,815 | 0.9% | 2,813 | 1.0% | 2 | 0.0% |
| Protein and Vitamin |  |  |  |  |  |  |
| Missing | 142,180 | 43.5% | 106,036 | 36.5% | 36,144 | 99.8% |
| Yes | 184,520 | 56.5% | 184,452 | 63.5% | 68 | 0.2% |
| **Presenting Symptoms** |  |  |  |  |  |  |
| Body Ache |  |  |  |  |  |  |
| No | 186,967 | 57.2% | 186,889 | 64.3% | 78 | 0.2% |
| Missing | 125,308 | 38.4% | 89,178 | 30.7% | 36,130 | 99.8% |
| Mild | 8,608 | 2.6% | 8,608 | 3.0% | 0 | 0.0% |
| Moderate | 5,147 | 1.6% | 5,143 | 1.8% | 4 | 0.0% |
| Severe | 670 | 0.2% | 670 | 0.2% | 0 | 0.0% |
| Breathing Difficulty |  |  |  |  |  |  |
| No | 182,148 | 55.8% | 182,074 | 62.7% | 74 | 0.2% |
| Missing | 125,308 | 38.4% | 89,178 | 30.7% | 36,130 | 99.8% |
| Mild | 11,593 | 3.5% | 11,589 | 4.0% | 4 | 0.0% |
| Moderate | 5,796 | 1.8% | 5,793 | 2.0% | 3 | 0.0% |
| Severe | 1,855 | 0.6% | 1,854 | 0.6% | 1 | 0.0% |
| Cough |  |  |  |  |  |  |
| No | 140,185 | 42.9% | 140,137 | 48.2% | 48 | 0.1% |
| Missing | 125,308 | 38.4% | 89,178 | 30.7% | 36,130 | 99.8% |
| Mild | 44,804 | 13.7% | 44,784 | 15.4% | 20 | 0.1% |
| Moderate | 15,130 | 4.6% | 15,116 | 5.2% | 14 | 0.0% |
| Severe | 1,273 | 0.4% | 1,273 | 0.4% | 0 | 0.0% |
| Diarrhea |  |  |  |  |  |  |
| No | 196,408 | 60.1% | 196,327 | 67.6% | 81 | 0.2% |
| Missing | 125,308 | 38.4% | 89,178 | 30.7% | 36,130 | 99.8% |
| Mild | 3,355 | 1.0% | 3,355 | 1.2% | 0 | 0.0% |
| Moderate | 1,500 | 0.5% | 1,499 | 0.5% | 1 | 0.0% |
| Severe | 129 | 0.0% | 129 | 0.0% | 0 | 0.0% |
| Fever |  |  |  |  |  |  |
| No | 179,245 | 54.9% | 179,178 | 61.7% | 67 | 0.2% |
| Missing | 125,308 | 38.4% | 89,178 | 30.7% | 36,130 | 99.8% |
| Mild | 16,973 | 5.2% | 16,965 | 5.8% | 8 | 0.0% |
| Moderate | 4,633 | 1.4% | 4,627 | 1.6% | 6 | 0.0% |
| Severe | 541 | 0.2% | 540 | 0.2% | 1 | 0.0% |
| Headache |  |  |  |  |  |  |
| No | 190,892 | 58.4% | 190,814 | 65.7% | 78 | 0.2% |
| Missing | 125,308 | 38.4% | 89,178 | 30.7% | 36,130 | 99.8% |
| Mild | 7,888 | 2.4% | 7,886 | 2.7% | 2 | 0.0% |
| Moderate | 2,326 | 0.7% | 2,324 | 0.8% | 2 | 0.0% |
| Severe | 286 | 0.1% | 286 | 0.1% | 0 | 0.0% |
| Loss of taste and smell |  |  |  |  |  |  |
| No | 159,345 | 48.8% | 159,284 | 54.8% | 61 | 0.2% |
| Missing | 125,308 | 38.4% | 89,178 | 30.7% | 36,130 | 99.8% |
| Mild | 24,585 | 7.5% | 24,578 | 8.5% | 7 | 0.0% |
| Moderate | 14,453 | 4.4% | 14,444 | 5.0% | 9 | 0.0% |
| Severe | 3,009 | 0.9% | 3,004 | 1.0% | 5 | 0.0% |
| Weakness |  |  |  |  |  |  |
| No | 145,154 | 44.4% | 145,095 | 49.9% | 59 | 0.2% |
| Missing | 125,308 | 38.4% | 89,178 | 30.7% | 36,130 | 99.8% |
| Moderate | 20,798 | 6.4% | 20,786 | 7.2% | 12 | 0.0% |
| Mild | 32,047 | 9.8% | 32,037 | 11.0% | 10 | 0.0% |
| Severe | 3,393 | 1.0% | 3,392 | 1.2% | 1 | 0.0% |
| **History of Travelling abroad** |  |  |  |  |  |  |
| No | 173,441 | 53.1% | 173,376 | 59.7% | 65 | 0.2% |
| Missing | 142,942 | 43.8% | 106,802 | 36.8% | 36,140 | 99.8% |
| Yes | 10,317 | 3.2% | 10,310 | 3.5% | 7 | 0.0% |
| **Hospitalization** |  |  |  |  |  |  |
| No | 299,021 | 91.5% | 262,823 | 90.5% | 36,198 | 100.0% |
| Yes | 27,581 | 8.4% | 27,567 | 9.5% | 14 | 0.0% |
| Missing | 98 | 0.0% | 98 | 0.0% | 0 | 0.0% |

S1 Table: Characteristics of the Study Population by availability of death outcome data
