# Supplementary material for: Risk factors for COVID-19 mortality among telehealth patients in Bangladesh: A prospective cohort study
Source: PLOS Glob Public Health. 2023 Jun 14;3(6):e0001971. doi: 10.1371/journal.pgph.0001971 (PMC10266619; doi:10.1371/journal.pgph.0001971)
Supplement: Attachment — Submitted filename: response to reviewer.docx [file pgph.0001971.s004.docx]

We are thankful to the editor and to the reviewers for the thoughtful comments of our manuscript. We have provided both the highlighted version and a clean version of the revised manuscript. As requested, below, we have provided a response to each editor and reviewer comment (*indicated in italics*) and identified the page and paragraph numbers where the changes (**indicated in bold**) have been made to the clean revised manuscript. Additionally, minor edits were made to the manuscript for consistency, clarification and simplification and are noted in the highlighted version of the revised manuscript.

**Editor’s comments:**

*1. A major concern is the lack of ethical approval in Bangladesh. Please explain why this is not required; these are highly sensitive data(sets).*

**Response:** We have obtained ethical approval from the institutional review board of icddr,b, (the International Center for Diarrhoeal Disease Research in Bangladesh). We have included the details in the method section **(page 5, paragraph 3).**

*2. 10% missing data on mortality outcomes is a very large proportion (much larger than the amount of deaths); the authors do mention this in the limitations. Seeing you will have most other data on these patients, have the authors done a comparison of the (baseline) characteristics to try to gauge the extent of the bias introduced by these missing data?*

**Response:** We compared the baseline characteristics of the participants with and without mortality data. Participants missing death outcome data have higher proportion of covariate data missing as for many of them physicians did not complete the initial assessment (99% missing doctors assessment data). These patients likely had mild cases of COVID which did not lead to a death outcome. Therefore, we anticipate that excluding these participants from analysis would have a small impact on the estimates of association with risk factors. The results are included as supplemental **Table 1 (Page 24).**

*2. The baseline table lacks information about physician assessment. Also, are there no data on hospitalisations? (And on what the physician's advice was, and whether this was followed up?)*

**Response:** We added the distribution of physician’s assessments in the baseline table. Proportion of hospitalization is added in Table 1 participant characteristics (**page 8**), Patients received follow up calls at scheduled intervals as mentioned in the methods section (**page 4, paragraph 1**). We do not have data on physician’s advice.

*3. The added value of the regression trees is not clear to me. Please spell out the implications for the Bangladesh situation (or remove entirely)*

**Response:** Thank you for this very helpful comment. The decision tree we present fills the gap for a handy decision-making algorithm that can be used by first-level health workers and clinicians for the initial assessments of COVID-19 patients during in-person and telehealth visits. We added a paragraph on the relevance of the regression tree for clinical decision making in the discussion section (**page 22, paragraph 3).**

*4. The methods lack clarity on merging of databases with death registry data*

**Response:** The databases were merged using unique identifier created by finding unique combinations of patients the mobile number, age, gender, and district of residence. Then the deidentified dataset was shared with the researchers. We included this detail in the method section **(page 5, paragraph 4).**

*5. The three different multivariable models based on the conceptual framework are difficult to understand (hence the question from reviewer 1, who clearly misunderstood that these were in fact multivariable models). I would suggest annotating the framework with the three models so it becomes more clear, and revise the text.*

**Response:** Thank you for this suggestion, we have annotated the conceptual framework and revised the text in the method section for clarity **(page 6, last paragraph).**

*6. I support the concern from reviewer 1 about the large number of missing data and how they were treated. Did you consider multiple imputation?*

**Response:** We did consider multiple imputation, but the characteristics of the missing data in our study are not suitable for multiple imputation. The missing responses are non-random, and we do not know which information is needed to infer the missing. Also, as we are interested in testing correlations between other variables in the dataset and running prediction models, therefore, we do not want to impose a correlation between variables by using multiple imputations. Due to the nature of the telehealth data, the amount of missing data is non-ignorable. As many variables of interest are categorical with multiple categories, and we do not know the cause of missingness or non-response, we created a missing class to retain the samples, and the missing class is potentially a meaningful marker of prediction.

*7. Please add rationale for the categorization in periods. The data only go up to June 2021, why is this the case? Please also give some context about the covid vaccination status; was the entire cohort unvaccinated?*

**Response:** The time periods are categorized considering the multiple peaks of cases observed, and predominant COVID-19 variants in Bangladesh. We describe this in detail in the methods section **(page 6, paragraph 2).** The vast majority of the cases were unvaccinated. Covid vaccination started in February 2021 by the end of June less than 4% of the population was vaccinated. We have described this in introduction and discussion **(page 3, paragraph 3, and page 36, paragraph 1).**

*8. Did you consider including the time periods in one of your models? The time period effect on mortality in covid waves is very strong.*

**Response:** Time period was included in the multivariable model( model 1) which included patient’s age, sex, location, period of the pandemic, socioeconomic status, comorbidities, and symptoms. Given the relationship of mortality during each time period and quality of care and care seeking pathway, we did not include time period in models 2 and 3 **(page 6, last paragraph).**

9. The number of mobile phones in the discussion is difficult to interpret without a total population size to give an indication of mobile phone penetration.

**Response:** We now state the proportion of Bangladeshi population with cellphone ownership and clarify that every household has at least one cellphone in the discussion section. **(page 22, last paragraph).**

**Reviewer #2:**

This manuscript examined the contributing risk factors of mortality due to Covid-19. The study does provide some novel findings on identifying a few important risk factors of mortality due to Covid-19 among the Bangladeshi population.

However, there are a few issues which need to be improved before publishing this manuscript.

*1. In the abstract the objective does not clearly state the problem for the research. Is this research only crucial because there is a lack of high-quality individual-level data?*

**Response:** Thank you for this comment. We now have stated the objectives clearly in the edited abstract **(page 2).**

*2. In the main manuscript, though the introduction states the problem for the research in the second paragraph it explained the methodology of this research. The introduction should consist of the existing background information and the objective and rationale of the study, not the methodology.*

**Response:** We have edited the introduction thoroughly, removed methods and added the rationale of the study. **(page 3, paragraphs 1 and 2).**

*3. The data is collected from the telehealth services which were self-reported by the patients. Did the researchers do any validity checks of the data to assess the accuracy of telemedicine assessments? If not, it should be listed as a limitation.*

**Response:** We accessed the data after the telehealth services ended. Therefore, we could not examine the validity of the self-reported symptoms. We now include this as a limitation of our research in the discussion section. **(page 22, last paragraph continued to page 23).**

*4. One of the most important findings of this research is physicians' assessment of the patients provides high predictability of mortality. But, how accurate this assessment is through telemedicine services? Its strength and limitations should be discussed in the discussion part.*

**Response:** We thank the reviewer for this comment. The accuracy of physician’s assessment was not validated against in-person clinical assessment. We have included this as another limitation of the study in the discussion section **(page 22, last paragraph continued to page 23).**

*5. Lastly, as both the incidence and mortality of Covid-19 is decreased and Bangladesh is successfully rolling out mass vaccination programmes, What is the implication of this study now? If there is any, then it should be discussed both in the introduction and discussion part.*

**Response:** We thank the reviewer for this very important comment. We have included in the introduction that vaccination started in February 2021 and by end of June, only 4% of the study population was vaccinated. **(page 3 , paragraph 3).** We also included in the discussion that these results are only generalizable to unvaccinated patients **(page 23, paragraph 1).**

**Reviewer 1**

Overall, the manuscript is well-written. However, I have some comments mentioned as follows:

*1. Throughout the manuscript, the context of low- and middle-income countries (LMIC) has been focused on. Therefore, it seems that the manuscript is based on LMICs though the manuscript is based on a database from Bangladesh, which should be revised to focus on the actual context, Bangladesh. We know that countries are classified into different categories based on income status. Despite belonging to the same income category by economic status, there is great variation among countries on other aspects including demographic, socio-economic, cultural, etc. Therefore, the context should be specific and the LMICs' context can be expressed in other ways and under some specific headings/ subheadings.*

**Response:** Throughout the revised manuscript, we now prioritize the literature from Bangladesh and clarified that the data is from Bangladesh only. We cite literature from other LMICs as there is limited data on risk factors available from Bangladesh.

*2. Missing data may present various problems such as, reduce statistical power, estimation bias, and reduce the representativeness of samples. In the result section of this manuscript, we are seeing that the percentage of missing data for many variables is huge (40-50%), which may lead to the mentioned issues. Thus, it is suggested to modify the study title to exclude the sample size information [290,488].*

**Response:** We have removed the number of participants from the title.

*3. Though the manuscript was prepared using secondary data, it would be suggested mentioning that whether the ethical issue is according to federal guidelines maintained at the time of information collection since the article is being published using this national-level data.*

**Response:** In the method section, we now include that the ethical approval for this study was obtained from icddr,b in Bangladesh and The New York State Psychiatric Institute. **(page 5, paragraph 3).**

*4. The authors failed to provide a framework for analyzing the data. No crosstab evaluation or statistical inferential tests or statistical analysis was performed which is a major drawback of the study. The authors should perform various analyses between the dependent and independent variables in their study. Also, why didn’t the author use a linear regression analysis for age instead of including age groups?*

**Response:** We now clarify in the method section that the analysis was informed by a conceptual diagram (supplemental Figure S1). We also clarify that the odds ratios presented throughout the manuscripts are adjusted odds ratios and the variables included in each model are now listed in the manuscript body and table titles.

Categorical age instead of linear age variable was used to examine the potential nonlinear relationship of age with mortality **(page 6, paragraph 1)**. Our results show that age effect was non-linear, very young children, and older patients had a higher risk of mortality from COVID-19 compared to the rest of the study population **(page 13, last paragraph)**.

*5. Why are so many age categories made?*

**Response:** We intend to address potential nonlinear effect of age by categorizing age. Our dataset is large, and we were able to retain enough participants within each age bin to infer the statistical significance. Five year age groups were chosen as similar age categories was used in analyses of age effects on COVID-19 mortality previous studies cited here and in the manuscript^1, 2^.

*6. Why only presented OR? Why is the AOR not presented?*

**Response:** the odds ratios presented throughout the manuscripts are adjusted odds ratios. We now list the variables included in the model in the manuscript body and the table title.

*7. Has any statistical been performed to quantify the effect of missing values on model accuracy?*

**Response:** The risk factors of primary interest are categorical. We generated a missing class for each variable to maximize the sample size and performed statistical analysis without imposing any assumptions about missingness. Thus, we did not exclude participants due to the missing data, and the effect of missing responses was quantified in the logistic regression compared to the reference group. We did not perform a separate analysis to evaluate the effect of missing values in model accuracy since they were not excluded in the analysis, and we treated the missing values as a separate category.

*8. An inconsistency in terms of age category has been seen between Table 1 (0-5, 5-10, …) and Table 2, 3, 4 (0-4, 5-9, …), which should be consistent across tables in a manuscript since there is a link between them and for the ease of understanding. So, the tables should be revised.*

***Response:*** Thank you for identifying this error. The age ranges are corrected in the revised manuscript.

*9. There are 8 administrative divisions in Bangladesh. Why a division (Mymensingh) was excluded? The name of the Chittagong division has been changed to Chattogram. So, it should be corrected throughout the manuscript. Moreover, it would be suggested to merge Chittagong S and Chittagong N into a single category for regional comparison.*

**Response:** We thank the reviewer for highlighting this omission. We have revised the analyses to include Mymensingh division. We initially kept Chittagong north and south separate as the mortality risk is different in the two parts of the division due potentially due to the difference in health system capacity and the quality of care provided. In the revised manuscript Chittagong is treated as a single division.

*10. Authors should start the discussion section with a short summary of what the main findings of their study instead of background information.*

**Response:** We have removed the background information and added a summary of the results in the beginning of the discussion **(page 34, paragraph 1)**.

*11. A contraction might be shown in the limitation paragraph. I would like to request to see the paragraph carefully once again.*

***Response:*** We did not fully understand this comment. However, we have revised the limitation paragraph to improve clarity.

*12. The authors need to present more statistical results in the abstract.*

***Response:*** We included statistical results in the revised abstract.

**References:**

1. Laxminarayan R, B CM, G VT, Arjun Kumar KV, Wahl B, Lewnard JA. SARS-CoV-2 infection and mortality during the first epidemic wave in Madurai, south India: a prospective, active surveillance study. Lancet Infect Dis. 2021 Dec;21(12):1665-1676. doi: 10.1016/S1473-3099(21)00393-5. Epub 2021 Aug 13. PMID: 34399090; PMCID: PMC8363227.
2. Elo IT, Luck A, Stokes AC, Hempstead K, Xie W, Preston SH. Evaluation of Age Patterns of COVID-19 Mortality by Race and Ethnicity From March 2020 to October 2021 in the US. *JAMA Netw Open.* 2022;5(5):e2212686. doi:10.1001/jamanetworkopen.2022.12686
